# Prevalence and impact of endocrine disorders in advanced metastatic cancer patients undergoing cancer‐directed therapy: A prospective observational study

**DOI:** 10.1002/cnr2.2011

**Published:** 2024-04-22

**Authors:** Gaurav Karna, Amit Sehrawat, Saurabh Karna, Ravi Kant, Deepak Sundriyal, Summi Karn, Dipesh Jha

**Affiliations:** ^1^ Department of Internal Medicine All India Institute of Medical Sciences Rishikesh India; ^2^ Department of Medical Oncology Haematology All India Institute of Medical Sciences Rishikesh India; ^3^ Department of Internal Medicine Kathmandu Medical College and Teaching Hospital Kathmandu Nepal; ^4^ Department of General Surgery All India Institute of Medical Sciences Rishikesh India; ^5^ Department of Geriatric Medicine All India Institute of Medical Sciences Rishikesh India

**Keywords:** Palliative care, Diabetes, Thyroid disorder, Screening, Advanced metastatic cancer

## Abstract

**Background:**

Noncommunicable diseases (NCDs) contribute significantly to global morbidity and mortality, with cancer being one of the leading causes. In this prospective observational study, we aimed to investigate the prevalence and impact of endocrine disorders, specifically diabetes and thyroid dysfunction, in patients with advanced metastatic cancer undergoing cancer‐directed therapy.

**Methods:**

Over 15 months, we recruited 100 histologically proven advanced metastatic cancer patients from the Department of Medical Oncology Haematology, All India Institute of Medical Sciences, Rishikesh, and conducted institutional‐based prospective observational study. All participants over 18 years of age, treatment‐naive, and potential candidates for systemic chemotherapy with an expected clinical survival of at least 6 months were included in the study. Patients with prior therapy, secondary neoplasms, and those unable to complete 3 months of palliative chemotherapy were excluded. Patients were assessed for diabetes and thyroid function at presentation, after 3 and 6 months of cancer‐directed standard therapy. These data were analyzed, processed, and presented as results.

**Results:**

The mean age of participants was 50.45 years, with a near‐equal distribution of males and females. At baseline, 10% of the study population had preexisting endocrine disorders (2% hypothyroidism, 8% diabetes). By the end of 6 months, the prevalence increased to 18%, with females being more affected. Notably, the prevalence of new‐onset endocrine disorders during cancer‐directed therapy was only 3% for diabetes and 4% for thyroid dysfunction.

**Conclusion:**

Analysis of sociodemographic and cancer‐related characteristics showed no significant association with changes in diabetic and thyroid status at 3 and 6 months. However, substance use, particularly smoking, was associated with an increased risk of diabetes development (*p* < .05). Cancer type and treatment regimen did not show statistically significant correlations with endocrine dysfunction.

**Implications:**

Our study highlights the importance of considering endocrine disorders in advanced metastatic cancer patients undergoing therapy. The prevalence of diabetes and thyroid dysfunction increased during cancer‐directed therapy, particularly in females. Careful monitoring and timely intervention are essential to improve the quality of life for these patients. Further research is warranted to explore the long‐term effects of cancer‐directed therapy on endocrine health and develop tailored management strategies for this vulnerable population.

## 1 INTRODUCTION

Noncommunicable diseases (NCDs) are the product of genetic, physiological, environmental, and behavioral alterations resulting in various comorbid states or mortality.^1^, ^2^ According to WHO, non‐communicable disease (NCD) kills 41 million people each year, which accounts for 74% of all global deaths; 17 million before age 70.86% of all premature death occurs in low‐ and middle‐income countries; the majority died because of cardiovascular disease (17.9 million per annum) followed by cancers (9.3 million), chronic respiratory diseases (4.1 million), and diabetes (2.0 million).^3^ In India, 63% of all deaths are estimated to be due to NCDs, and cancer was one of the leading causes (9%).^4^

GLOBOCAN‐2020, produced by the International Agency for Research on Cancer (IARC) shows 19.3 million total new cases of cancer and 10 million cancer‐related deaths.^5^ In India's context from 1990 to 2016, there is a 90.9% increase in the total DALY and a 112.8% increase in total death due to all cancers. The burden of cancer has been increasing in the last two decades rapidly; 28.2% from 1990 to 2016.^6^

Globally, the prevalence of diabetes is about 27% in people over 65 years.^7^ Likewise, the prevalence of thyroid disease in a diabetic is about 10.8%, with hypothyroid and subclinical hypothyroid being 30% and 50%, respectively.^8^ Globally, the current 6.6% of thyroid dysfunction is expected to increase with advancing age. Despite the high prevalence of these NCDs, the screening is limited to the targeted population.

“Advance cancer” is often used synonymously for metastatic cancer. However, the National Cancer Institute defines advanced cancer as any cancer that could not be cured or contained by treatment.^9^, ^10^ As a result of evolving medical science, cancer‐directed therapies have been a cornerstone for advanced cancer. Although the incidence of cancer is increasing worldwide, cancer‐related death is consistently dropping because of advances in cancer therapeutics. However, cancer survivors are at potential risk of developing some therapy‐related late effects.^11^ Although cancer therapy in children, adolescents, and adults has been associated with metabolic syndromes and an increased risk of diabetes mellitus and cardiovascular disease, they are often overlooked and inadequately addressed.^12^ Evidence has grown in the last few decades, revealing a high cumulative incidence of chronic therapy‐associated morbidity in long‐term cancer survivors in support of therapy‐related side effects.^13^ Although the overall survival in childhood cancer has dramatically increased over a few decades, these improvements in survival rates have been achieved at the expense of various late complications primarily involving the endocrine system.^14^

Based on various studies worldwide, endocrine disorders are rising as a major challenge in cancer patients on cancer therapy. Most have a functional alteration of hypothalamic‐pituitary, thyroid, parathyroid, adrenal, and gonadal regulation. Occasionally, bone and metabolic complications are also seen.^15^ Immunotherapy for metastatic solid tumors and melanoma, Cytarabine, and daunorubicin used for the induction phase in Acute leukemia has reported transient thyroid dysfunction.^15^, ^16^, ^17^ Similarly, the increasing risk of hyperthyroidism in Hodgkin's disease was seen after radiation exposure.^17^ Unlike thyroid disorder, Tyrosine kinase inhibitors used in CML and radiation therapy in breast cancer were associated with an increased number of denovo diabetes by a transient decline in β‐cell function.^18^ Nowadays, because of the unclear but proven association between thyroid disorders and the use of immune checkpoint inhibitors (like PD‐1 and PD‐L1), their long‐term association is becoming a new study dimension.^19^ The mechanisms of endocrine dysfunction with most of the cancer therapy and/or cancers are poorly understood. Also, there is a lack of evidence on cancer in adulthood due to the tendency to follow for 5 years of recurrence‐free survival under conventional surveillance. Therefore, evidence‐based recommendations for overall long‐term medical surveillance of these adult cancer survivors do not currently exist.^20^

To the best of our knowledge, there is a paucity of data on the impact of palliative chemotherapy on the endocrinological profile of patients with advanced metastatic cancer. The main aim of this study is to breach the gap and identify whether cancer‐directed therapy, which may be immunotherapy, cytotoxic drugs, or targeted therapy, causes any changes in the endocrinological profile, mainly the diabetic and thyroid status of the patient living with advanced metastatic cancer.

## 2 SUBJECTS AND METHODS

### 2.1 Sample Design

**FIGURE 1:**
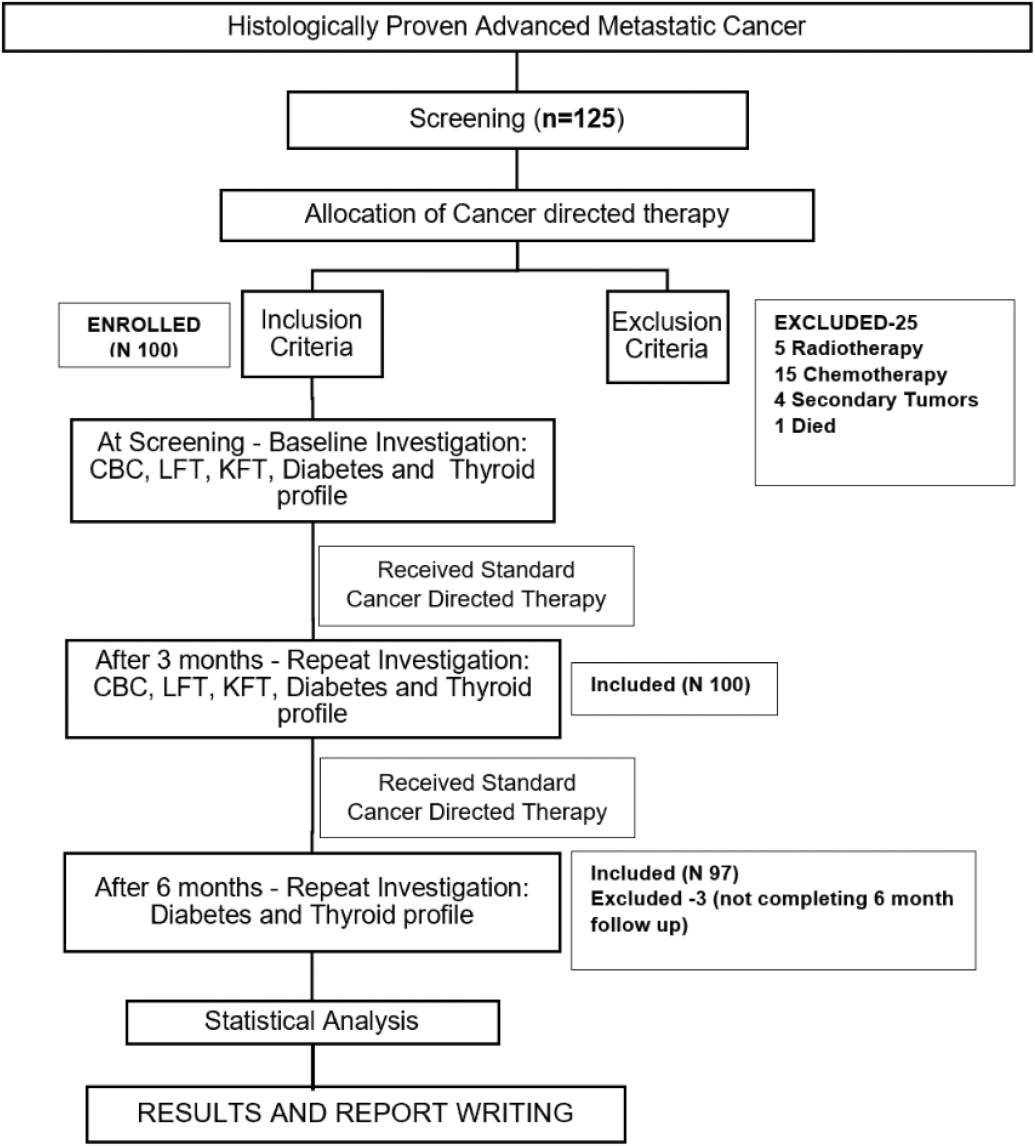
The study flow diagram.

A prospective observational study was conducted at outpatient and inpatient services at the Department of Medical Oncology Hematology, All India Institute of Medical Sciences, Rishikesh over 15 months (May 2021–July 2022) after acquiring the Institute's ethical clearance in May 2021 (Figure 1 shows a study flow diagram).

After approval from the institutional ethics committee and written informed consent, all patients meeting the inclusion criteria were enrolled in the study. Screening test for diabetes and thyroid status were done in 3 settings (baseline, after 3 months, and after 6 months). As per the standard institute protocol, all these patients were subjected to standard cancer‐directed therapy (palliative intent). Almost all candidates received adjunct medications (like steroids, antiemetics, etc.).

All the candidates with deranged blood sugar levels or HbA1c were categorized into diabetes, prediabetes, and non‐diabetes based on the American Diabetes Association (ADA) definition. The criteria for diagnosing diabetes and prediabetes were as follows: fasting plasma glucose ≥125 mg/dL or 2‐h plasma glucose ≥200 mg/dL during OGTT or HbA1c ≥6.5% for diabetes, and fasting plasma glucose between 100 and 125 mg/dL or 2‐h plasma glucose 75 g OGTT between 140 and 199 mg/dL or HbA1c > 5.7% for prediabetes.

All the patients with deranged Thyroid function tests were classified into either of the following groups based on the ATA/AACE guidelines. The criteria for defining hypothyroidism, subclinical hypothyroidism, and hyperthyroidism were as follows: TSH level > 4.5 mIU/L with fT4 below the lower reference value for hypothyroidism; TSH level > 4.5 mIU/L with fT4 within the reference range for subclinical hypothyroidism; and TSH level < 0.64 mIU/L with fT4 above the upper reference value for hyperthyroidism.

### 2.2 Sample Size

All inpatient and outpatient patients presenting at the Department of Medical Oncology Haematology eligible for inclusion and exclusion criteria during the time frame of 15 months (from May 2021 to July 2022) were subjected to the study.

### 2.3 Primary & Secondary Outcome

Determining diabetes and thyroid status in advanced cancer patients and analyzing the changes after standard cancer‐directed therapy were primary objectives. The secondary objectives were to study the correlation between changes in these endocrine profiles with the type of cancer and type of therapy received. After obtaining informed written consent, all the candidates were screened for diabetes and thyroid profiles.

### 2.4 Inclusion Criteria

All patients over 18 years who were histologically proven advanced metastatic cancer patients and potential candidates for systemic chemotherapy with an expected clinical survival of at least 6 months were included in the study.

### 2.5 Exclusion Criteria

All those patients who were pregnant or had secondary neoplasms received prior cancer‐directed therapy, or those who did not receive at least 3 months of chemotherapy (palliative intent) were excluded from the study.

### 2.6 Statistical analysis

All collected data were entered, cleaned, and coded in MS Excel spreadsheet. Data analysis was performed using SPSS v20.0. Sociodemographic characters (age, gender, etc.) of the study population were presented as means, median, standard deviation, and interquartile range. Cancer‐related characteristics (type, therapy) were expressed as percentages. All the parametric data were compared using the paired *t*‐test and ANOVA. And the non‐parametric data were compared using the Chi‐square test (Pearson’s, Fisher's exact, or Likelihood ratio). Error bars are used to show the change in trends in the graphical presentation. Considering the confidence level of 95%, the *p*‐value at <.05 was statically significant.

### 2.7 Ethical Clearance

The ethical clearance for conducting the study was received from the Institutional Ethical Committee in May 2021 (AIIMS/IEC/21/296).

## 3 RESULTS

The mean age of participants was 50.45 years (SD =1 4.612). The age ranged from 18‐ 80 years (median age ‐ 50.5 years), with an almost equal proportion of males (51%) and females (49%). At the time of diagnosis, 2% had hypothyroid, and 8% had hyperthyroid. Table 1 shows the baseline sociodemographic characteristics of eligible participants concerning their baseline endocrine profile.

In the study, the most common malignancy was gynecological (36%), followed by Lungs (23%), Genitourinary (16%), and Gastrointestinal (17%). Most received platinum‐based chemotherapy (82%), followed by Tyrosine kinase inhibitors (4% received sunitinib and Erlotinib), and the remaining 10% received other cancer‐directed therapies. The study shows that endocrine disorders are seen in ovarian cancer, followed by lung cancer (excluding breast cancer). Although endocrine disorders are common with Paclitaxel‐carboplatin followed by AIM regimen, they do not reflect a statically significant relation.

The association between the changes from baseline thyroid status and diabetic status were compared at follow‐ups of 3 months and 6 months, respectively. This study shows there is no significant association between changes in endocrine status with respect to age, marital status, and cancer type. Table 2 shows the conversion of diabetes and thyroid dysfunction to normal was more in those who do not use any substance and females, respectively (p<0.05) on 6 months of follow‐up.

**FIGURE 2:**
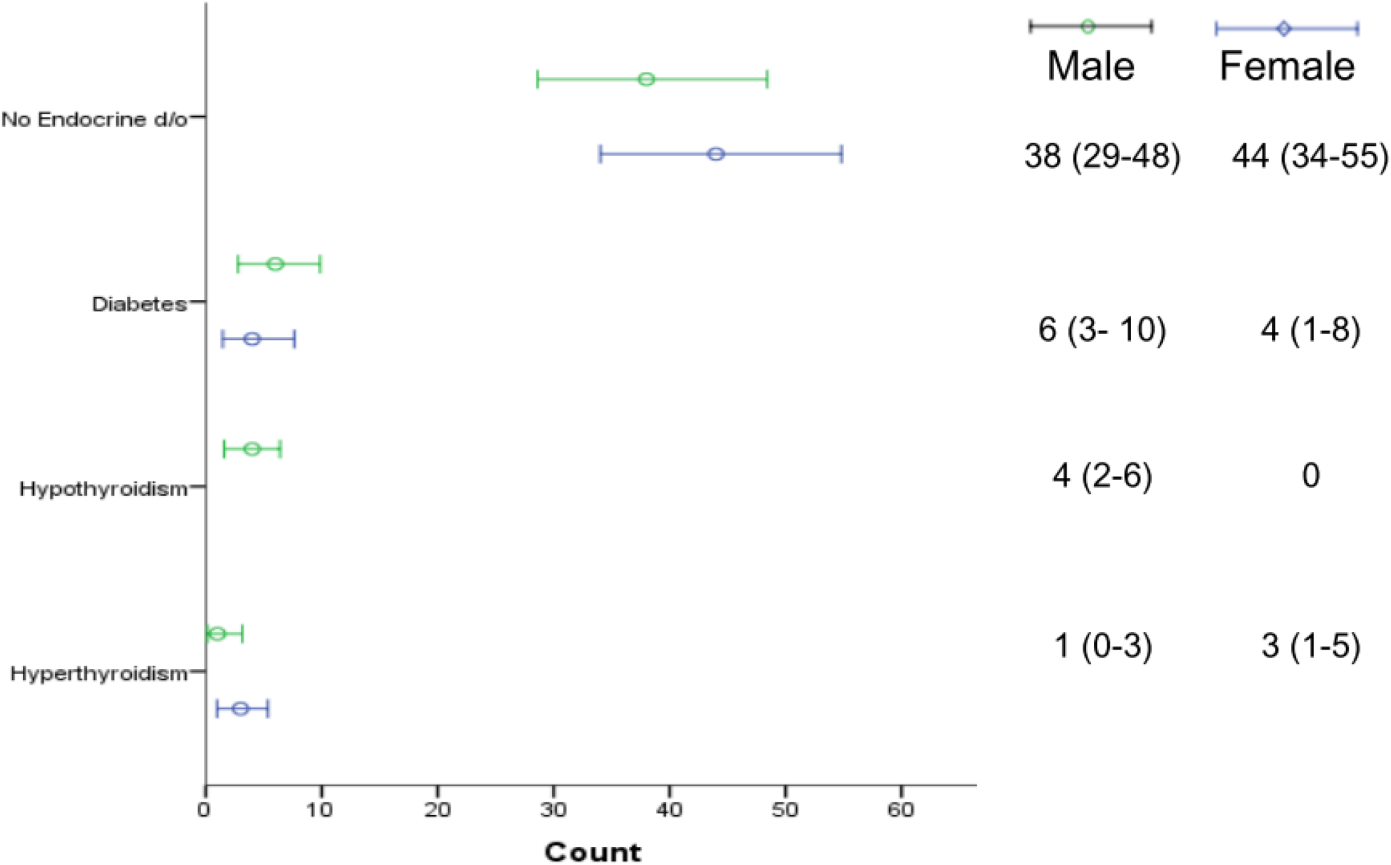
Prevalence of selected endocrine disorder at last follow‐up visit, by gender.

The prevalence of these endocrine disorders was 18% during 6 months follow‐up, of which 11% were female and 7% were male. Out of 100, 10% had either of two endocrine disorders (2% had thyroid dysfunction and 8% had diabetes) at the time of diagnosis of cancer. By the end of the study, we observed 3% and 4% of enrolled patients newly developed diabetes and thyroid dysfunction, respectively. Table 3 demonstrates the change in the diabetic profile and thyroid status through their 6 months of follow‐up. There were no significant changes in the average lab parameters (TSH, fT3, fT4, Fasting, and PP glucose) of the study population except for HBA1c (p<0.05). Although everyone received steroids as an adjunct, there was no increase in prediabetic cases (16% at baseline compared to 13% at 6 months). (Table 4, Figure 2).

**TABLE 1:** Sociodemographic profile and cancer‐related characteristics of the study population.

	With endocrine disorder (*n* (%))	Without endocrine disorder (*n* (%))
Gender		
Female	3 (3)	48 (41)
Male	8 (8)	41 (41)
Marital status		
Married	11 (11)	86 (86)
Unmarried	0 (0)	3 (3)
Addictions		
Smoking	2 (2)	23(19)
Alcohol	1 (0)	0 (0)
Tobacco	1 (0)	1 (1)
None	9 (9)	65 (65)
Religion		
Hindu		
Muslim	10 (10)	67 (67)
Christian	1 (1)	16 (16)
Age (years)		
Youth (18–30)	0 (0)	6(6)
Adults (30–60)	8 (8)	58 (58)
Seniors (over 60)	3 (3)	25 (25)
Site of cancer		
Ovary	6 (6)	27 (27)
Lungs	2 (2)	21 (21)
Urinary bladder	2 (2)	9 (9)
Others	1 (1)	32 (32)
Treatment regimen		
Pacli‐Carbo	5 (5)	29 (29)
AIM	3 (3)	2 (2)
MVAC	2 (2)	3 (3)
Eto‐Cis	2 (2)	6 (6)
Others	6 (6)	42 (42)

Abbreviations: AIM, Adriamycin, Ifosphamide, Mesna; Eto‐Cis, Etoposide and Cisplatin; MVAC, Methotraxate, Vinblastine, Adriamycin, Cisplatin; Pacli‐Carbo, Paclitaxel and Carboplatin.

**TABLE 2:** Association of change in the diabetic and thyroid status with various characteristics over 3 and 6 months.

Characteristics	*p* Value (statically significant if *p* value < .05)			
	At the end of 3 months		At the end of 6 months	
	Changes in thyroid status	Changes in diabetes status	Changes in thyroid status	Changes in diabetes status
Age	0.448	0.908	0.832	0.245
Gender	0.548	0.566	0.031	0.155
Marital status	0.171	0.793	0.288	0.426
Substance use	0.224	0.693	0.349	0.030
Cancer type	0.134	0.278	0.192	0.190

**TABLE 3:** Change in the endocrine profile of the study population with respect to time during the course of palliative chemotherapy.

	Mean (SD)			*p*‐Value
	Baseline	3 months	6 months	
fT3	3.42 (13.55)	2.41 (6.01)	2.73 (1.2)	.612
fT4	2.76 (3.18)	2.73 (1.57)	2.74 (1.13)	.982
TSH	4.66 (11.71)	3.57 (2.26)	2.68 (1.11)	.168
HBA1c	5.48 (0.88)	5.37 (0.68)	5.14 (0.68)	**<.01**
Fasting	88.61 (13.53)	88.29 (13.84)	88.66 (7.75)	.960
Post prandial	122.82 (24.97)	120.34 (24.88)	123.75 (15.47)	.485

*Note*: Bold value indicates how the endocrine profile changes in study population with respect to time after palliative chemotherapy.

**TABLE 4:** Summary table with distribution of endocrine disorder with respect to time during the course of palliative chemotherapy.

	Total	Baseline *n* (%)	After 3 months *n* (%)	After 6 months *n* (%)
Thyroid status	Hyperthyroid	1 (1)	3 (3)	0 (0)
	Euthyroid	89 (89)	93 (93)	91 (91)
	Subclinical hypothyroid	8 (8)	3 (3)	5 (5)
	Hypothyroid	2 (2)	1 (1)	1 (1)
Diabetes status	Non‐diabetic	76 (76)	73 (73)	80 (80)
	Pre‐diabetic	16 (16)	19 (19)	13 (13)
	Diabetic	8 (8)	8 (8)	4 (4)

## 4 DISCUSSION

The prevalence of noncommunicable diseases (NCDs) is increasing globally, with diabetes and cancer being significant contributors to morbidity and mortality.^21^ In this study, we focused on understanding the impact of cancer‐directed therapy on the endocrinological profile of patients with advanced metastatic cancer. Our findings highlight the substantial prevalence of endocrine disorders, particularly diabetes and thyroid dysfunction, in this vulnerable population.

Previous studies have shown an increased risk of late complications in cancer survivors, with endocrinopathies being more common among childhood cancer survivors.^22^ However, evidence on endocrine disorders in adult cancer survivors remains limited, and our study aimed to bridge this gap. We observed a higher prevalence of endocrine disorders among females compared to males, consistent with other investigations.^14^

The association between diabetes and cancer has been extensively studied, revealing shared risk factors such as substance use, including alcohol and tobacco smoking.^23^, ^24^, ^25^ Our study also found a correlation between non‐smokers and earlier normalization of diabetic and thyroid parameters during cancer‐directed therapy.

Moreover, cancer‐related therapy, including high‐dose alkylating agents and irradiation, has been implicated in causing endocrine disorders.^12^, ^26^ Platinum‐based compounds and immunotherapy regimens, such as AIM (Adriamycin, Ifosphamide, Mesna) and IL2‐based immunotherapy, have also been associated with endocrine dysfunction.^27^ In our study, we observed a higher prevalence of endocrine disorders in patients receiving platinum‐based and AIM regimens.

The most common cancers associated with secondary diabetes include lung, breast, and colorectal cancers.^23^ Similarly, thyroid dysfunction, including hyperthyroidism and Graves' disease, has been reported in patients receiving interferon alfa therapy.^16^ In our study, we identified transient thyroid dysfunction during cancer‐directed therapy, affecting 8% of the study population, with covert subclinical hypothyroidism being the most prevalent.

Steroid use is common in cancer therapeutics for various purposes, including managing cancer pain, therapy‐associated side effects, and anti‐inflammatory actions. However, steroids can induce hyperglycaemia by altering beta cell function and insulin sensitivity.^28^, ^29^ In our study, almost all patients received steroids as an adjuvant during chemotherapy, and we observed a transient increase in prediabetes prevalence, which later normalized during the 6‐month follow‐up.

Our present study shows that NCDs like diabetes and thyroid disorders contribute to morbid states in metastatic cancer patients, which need to be screened and addressed subsequently. The steroid use in cancer patients not only increases compliance and efficacy of treatment is associated with transient fluctuation in diabetes and thyroid function during treatment. Despite the significant findings, our study has some limitations. It was conducted in a single center, limiting the generalization of results. Future multicentric studies are warranted to establish a causal temporal relationship between cancer, its therapeutic interventions, and endocrine disorders. Moreover, long‐term follow‐up is essential to confirm the association between specific drugs, cancer types, and endocrine disorders.

## 5 CONCLUSION

This study emphasizes the importance of considering comorbidities like diabetes and thyroid dysfunctions in advanced metastatic cancer patients. The prevalence of these endocrine disorders is notably high among ovarian and lung cancer patients, while breast cancer patients show lower rates. The use of steroids as an adjunct during chemotherapy did not increase diabetic and prediabetic cases, supporting their role in improving therapy compliance and reducing side effects.

Addressing these comorbidities can enhance the holistic approach to patient care and improve the quality of life for those facing terminal illness. However, the study's single‐center nature limits generalization, necessitating larger multicenter studies to establish causal relationships between cancer, therapy, and endocrine disorders. Despite limitations, this research contributes valuable evidence, filling a critical gap in the existing literature. Managing these comorbidities promptly can lead to better care and outcomes for patients with advanced metastatic cancer.

## AUTHOR CONTRIBUTIONS

**Gaurav Karna:** Conceptualization (lead); data curation (lead); formal analysis (lead); investigation (lead); methodology (lead); project administration (lead); resources (equal); software (equal); supervision (lead); validation (equal); visualization (equal); writing – original draft (lead); writing – review and editing (lead). **Amit Sehrawat:** Conceptualization (lead); data curation (lead); formal analysis (equal); investigation (lead); methodology (equal); project administration (equal); resources (equal); supervision (lead); visualization (equal); writing – review and editing (equal). **Saurabh Karna:** Conceptualization (lead); data curation (equal); formal analysis (lead); methodology (equal); project administration (equal); resources (lead); software (lead); validation (equal); visualization (equal); writing – original draft (lead); writing – review and editing (lead). **Ravi Kant:** Conceptualization (equal); methodology (equal); project administration (equal); supervision (equal); validation (equal). **Deepak Sundriyal:** Conceptualization (equal); investigation (equal); methodology (equal); project administration (equal); resources (equal); supervision (equal). **Summi Karn:** Conceptualization (equal); formal analysis (equal); investigation (equal); project administration (equal); resources (equal); software (equal); validation (equal); writing – original draft (equal); writing – review and editing (equal). **Dipesh Jha:** Investigation (equal); resources (equal); validation (equal); visualization (equal); writing – original draft (equal); writing – review and editing (equal).

## CONFLICT OF INTEREST STATEMENT

The authors have stated explicitly that there are no conflicts of interest in connection with this article.

## Data Availability

Data sharing is not applicable to this article as no new data were created or analyzed in this study.
